# Biomarkers of cetuximab resistance in patients with head and neck squamous cell carcinoma

**DOI:** 10.20892/j.issn.2095-3941.2019.0153

**Published:** 2020-02-15

**Authors:** Olivia Leblanc, Sophie Vacher, Charlotte Lecerf, Emmanuelle Jeannot, Jerzy Klijanienko, Frédérique Berger, Caroline Hoffmann, Valentin Calugaru, Nathalie Badois, Anne Chilles, Maria Lesnik, Samar Krhili, Ivan Bieche, Christophe Le Tourneau, Maud Kamal

**Affiliations:** ^1^Department of Genetics, Curie Institute, PSL Research University, Paris 75005, France; ^2^Department of Drug Development and Innovation (D3i), Curie Institute, PSL Research University, Paris 75005, France; ^3^Department of Pathology, Curie Institute, PSL Research University, Paris 75005, France; ^4^Department of Biostatistics, Curie Institute, PSL Research University, Paris 75005, France; ^5^Department of Surgery, Curie Institute, PSL Research University, Paris 75005, France; ^6^INSERM U932 Research Unit, Paris 75005, France; ^7^Department of Radiotherapy, Curie Institute, PSL Research University, Paris 75005, France; ^8^EA7331, Faculty of Pharmaceutical and Biological Sciences, Paris Descartes University, Paris 75005, France; ^9^INSERM U900 Research Unit, Curie Institute, Paris 75005, France

**Keywords:** Head and neck squamous cell carcinoma, cetuximab, biomarker, PIK3CA, RAS

## Abstract

**Objective:** In patients with head and neck squamous cell carcinoma (HNSCC), cetuximab [a monoclonal antibody targeting epidermal growth factor receptor (EGFR)] has been shown to improve overall survival when combined with radiotherapy in the locally advanced setting or with chemotherapy in first-line recurrent and/or metastatic (R/M) setting, respectively. While biomarkers of resistance to cetuximab have been identified in metastatic colorectal cancer, no biomarkers of efficacy have been identified in HNSCC. Here, we aimed to identify biomarkers of cetuximab sensitivity/resistance in HNSCC.

**Methods:** HNSCC patients treated with cetuximab at the Curie Institute, for whom complete clinicopathological data and formalin-fixed paraffin-embedded (FFPE) tumor tissue collected before cetuximab treatment were available, were included. Immunohistochemistry analyses of PTEN and EGFR were performed to assess protein expression levels. *PIK3CA* and *H/N/KRAS* mutations were analyzed using high-resolution melting (HRM) and Sanger sequencing. We evaluated the predictive value of these alterations in terms of progression-free survival (PFS).

**Results:** Hot spot activating *PIK3CA* and *KRAS/HRAS* mutations were associated with poor PFS among HNSCC patients treated with cetuximab in the first-line R/M setting, but not among HNSCC patients treated with cetuximab in combination with radiotherapy. Loss of PTEN protein expression had a negative predictive value among HNSCC patients treated with cetuximab and radiotherapy. High EGFR expression did not predict cetuximab sensitivity in our patient population.

**Conclusions:** Hot spot activating *PIK3CA* and *RAS* mutations predicted cetuximab resistance among HNSCC patients in the first-line R/M setting, whereas loss of PTEN protein expression predicted resistance to cetuximab when combined to radiotherapy.

## Introduction

Head and neck squamous cell carcinoma (HNSCC) is the most common cancer of the head and neck, and the seventh most common cancer overall, affecting around 600,000 patients per year worldwide^1^. The main risk factors are smoking and alcohol consumption, which are responsible for the majority of HNSCC cases in the oral cavity, pharynx, and larynx. Human papillomavirus (HPV) was also recently identified as a risk factor for oropharyngeal cancer^2^. Despite advances in multimodal therapy, the overall 5-year survival rate of patients with locally advanced HNSCC remains poor around 40%–50%. Overall survival in the locally advanced setting has been shown to be improved by adding cetuximab to radiation therapy^3^. In the first-line recurrent and/or metastatic (R/M) setting, cetuximab has been shown to improve overall survival when combined with chemotherapy^4^. Cetuximab is therefore widely used in both the locally advanced and recurrent and/or metastatic settings^5–8^. Despite these improvements, only a minority of patients in these settings benefit from cetuximab treatment. Thus, it is critical to identify predictive biomarkers of sensitivity/resistance to cetuximab in HNSCC.

Epidermal growth factor receptor (*EGFR*) amplifications were not shown to predict cetuximab efficacy in combination with chemotherapy^9^ and other mechanisms of resistance to EGFR inhibitors have been reported including the activation of downstream signals such as *via KRAS* and *PIK3CA* mutations^10^. A gene signature based on more than 509 differentially expressed genes was reported to be predictive of response to cetuximab in HNSCC^11^.

In 2015, The Cancer Genome Atlas reported that the molecular landscape of HNSCC includes identified mutations in various oncogenes [*PIK3CA* (21%) and *HRAS* (4%)] and tumor suppressor genes [*TP53* (72%), *CDKN2A* (22%), *FBXW7* (5%), *KMT2D* (*MLL2*) (18%), and *PTEN* (2%)]^12^. Phosphoinositide 3-kinases (PI3Ks) play key regulatory roles in multiple cellular processes, including cell survival, proliferation, and differentiation. A broad range of human cancers exhibit frequent alterations in many components of the PI3K/AKT pathway. *PIK3CA* mutations/amplifications and *PTEN* loss, respectively, occur in around 34% and 12% of HNSCC cases^13^.

In metastatic colorectal cancer, *KRAS* mutations were reported to predict resistance to cetuximab^14,15^. In cervical cancer patients treated with cetuximab and radiotherapy in a curative intent, downstream PI3K/AKT pathway activation was associated with a resistance to cetuximab^16^.

In the present study, we aimed to identify the predictive biomarkers of response to cetuximab by analyzing EGFR and PTEN expression, and *PIK3CA* and *RAS* mutations.

## Patients and methods

### Patients and samples

This study included HNSCC patients treated with cetuximab at the Curie Institute, from whom complete clinicopathological data and formalin-fixed paraffin-embedded (FFPE) tumor tissues collected before the cetuximab initiation were available. Disease staging was based on the 7th revised edition (2010) of the American Joint Committee on Cancer (AJCC). All patients were informed that their tumor samples might be used for scientific purposes and had the opportunity to decline. This study was approved by the Internal Review Board of the Curie Institute, and was conducted in accordance with the ethical principles of the Declaration of Helsinki.

### DNA extraction

From FFPE tissues, we obtained 6 tissue sections (6-µm thick), and a 7th tissue section that was stained with hematoxylin-eosin. The tumor-rich areas were macrodissected using a single-use blade, and the samples underwent proteinase K digestion in a rotating incubator at 56 °C for 3 days. DNA was extracted using the Nucleospin® 8 Tissue kit (Macherey-Nagel, GmbH & Co. KG, Germany).

### *RAS* and *PIK3CA* mutations

To screen for mutations, high-resolution melting (HRM) primers were designed for *HRAS* (exons 2 and 3), *NRAS* and *KRAS* (exons 2–4), and *PIK3CA* (exons 9 and 20). Polymerase chain reaction (PCR) for HRM analysis was performed using the fluorescent DNA-intercalating dye LC green (Idaho Technology), in a 384-well plate using a LightCycler480® (Roche). The reaction mixture had a final volume of 15 µL, and contained LC green, UDP Glycosylase (Roche), and Roche Master Mix (Roche). The reaction conditions were as follows: 40 °C for 10 min, 95 °C for 10 min; 50 cycles of 95 °C for 15 s, 55–65 °C for 15 s, and 72 °C for 25 s; followed by 95 °C for 1 min, and then melting from 65 °C to 95 °C, rising 0.02 °C per s. All samples were tested in duplicate. HRM analysis was performed using Genescan software (Roche). All samples, including the wild-type exons, were plotted on a differential plot graph according to their melting profiles. When an abnormal HRM curve was suspected, the samples were sequenced using the Sanger sequencing approach.

### HPV genotyping

HPV status was assessed at the Pathology Department, where HPV typing was conducted using total DNA isolated from FFPE samples of HNSCC tumors. Real-time PCR was performed with Sybr® Green and specific primers for HPV16, 18, and 33, using a 7900HT Fast Real-Time PCR System (Applied Biosystems). HPV L1 amplicons from HPV16-, 18-, and 33-negative samples were sequenced by the Sanger method using the GP6+ primer. HPV type identification was performed *via* sequence alignment with HPV reference sequences using the NCBI nucleotide blast program (http://blast.ncbi.nlm.nih.gov/Blast.cgi).

### Immunohistochemistry

Immunohistochemical assays using EGFR antibodies (monoclonal mouse, 31G7, Trypsin; InVitrogen) and PTEN antibodies (monoclonal, PTEN amino acids 321–336; Zymed® Laboratories) were performed at the Pathology Department. From the FFPE samples of HNSCC tumors, 3-µm sections were cut, and were deparaffinized and rehydrated through a series of xylene and ethanol washes. All immunostaining processes were performed using a LEICA (BOND III) automated immunostaining device. IHC was performed in some samples exhibiting no intensity (0) and high intensity (3) for EGFR and PTEN, and a score (0%–100%) for the expression of these proteins was established. Membrane EGFR staining was taken into account. For PTEN, an internal control was a positive expression of PTEN in the stromal fibroblasts. A normal prostate tissue was used for an external control in every manipulation. Interpretation of the staining determined the percentage of tumoral positive cells, the intensity of the staining on a three-tiered scale (form 1 to 3) as well as the good quality of the external control and the presence of the internal control. Nuclear expression of PTEN was not considered in the framework of the interpretation of PTEN expression levels. Samples were rated as having high or low expression of EGFR and PTEN using a semi-quantitative histological score: low EGFR = intensity ≤ 2 or protein expression ≤ 80%; high EGFR = intensity > 2 and protein expression > 80%; low PTEN = intensity ≤ 1 and protein expression ≤ 20%; high PTEN = intensity >1 and protein expression > 20%.

### Statistical analysis

We recorded patients’ characteristics and survival data up to June 2016. Categorical variables were compared using a Chi-square (χ^2^) test, with Yates’s correction when appropriate. Patients alive at the end of follow-up were censored at the date of their last visit. Progression-free survival (PFS) was defined as the period from the first day of cetuximab therapy to the date of first disease progression or death from any cause. Survival distributions were estimated by the Kaplan–Meier method, and compared using the log-rank test. The univariate Cox proportional hazard model was used to evaluate the predictive values of clinical and molecular markers for PFS. Results are presented as hazard ratio (HR) and 95% confidence interval (CI). A *P* value of < 0.05 was considered to be significant. Analyses were performed using R software version 3.3.2 (R Core Team 2016).

## Results

### Patient and tumor characteristics

We analyzed tumor samples from 118 HNSCC patients who were treated with cetuximab between 2006 and 2015. Three patients were lost to follow-up and thus excluded from the analyses. **Table 1** summarizes the clinical, biological, and pathological characteristics of the 115 remaining HNSCC patients. Median follow-up was 54.1 months (range: 3.2–92.5 months). The median age at diagnosis was 60 years. Patients were mainly males. Most patients were smokers. Fifteen patients (13%) had HPV-positive tumors. Of the 115 patients, 77 (67%) were treated with cetuximab in the first-line R/M setting (group 1), and 38 (33%) were treated with cetuximab and radiotherapy in the locally advanced setting (group 2) (**Supplementary Tables S1 and S2**). Only patients who were not eligible for high-dose cisplatin were treated with cetuximab in combination with radiotherapy in the locally advanced setting. These two groups showed similar distributions of clinical, biological, and pathological parameters, except for age and HPV status (**Supplementary Table S3**). Compared to group 1, patients from group 2 were older and more frequently had HPV-positive tumors.

### Predictive value of *PIK3CA* and *RAS* mutations on cetuximab efficacy

#### Whole population

**Figure 1:**
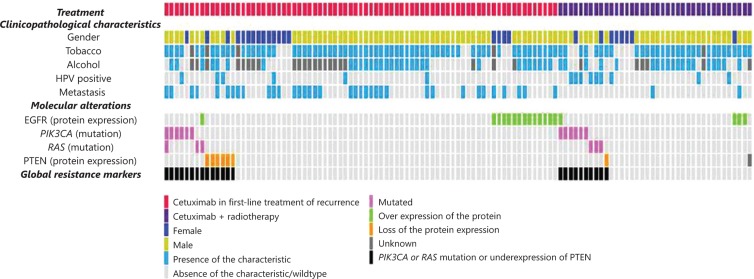
Clinical, biological, pathological, and molecular characteristics of 115 HNSCC patients.

Of the 115 patients, 17 (14.8%) had *PIK3CA* and/or *RAS* gene mutations, including 12 (10.4%) with a *PIK3CA* mutation, 2 (1.7%) with a *KRAS* mutation, and 4 (3.5%) with a *HRAS* mutation. One patient had both a *PIK3CA* mutation and a *HRAS* mutation (**Table 2**, **Figure 1**). All tumors exhibited a wild-type *NRAS* gene. **Supplementary Table S4** presents details of the mutational profiles. In the whole population, *PIK3CA*, *KRAS*, and *HRAS* mutations did not correlate with PFS (**Table 2**).

#### Group 1: cetuximab given in the first-line recurrence setting

**Figure 2:**
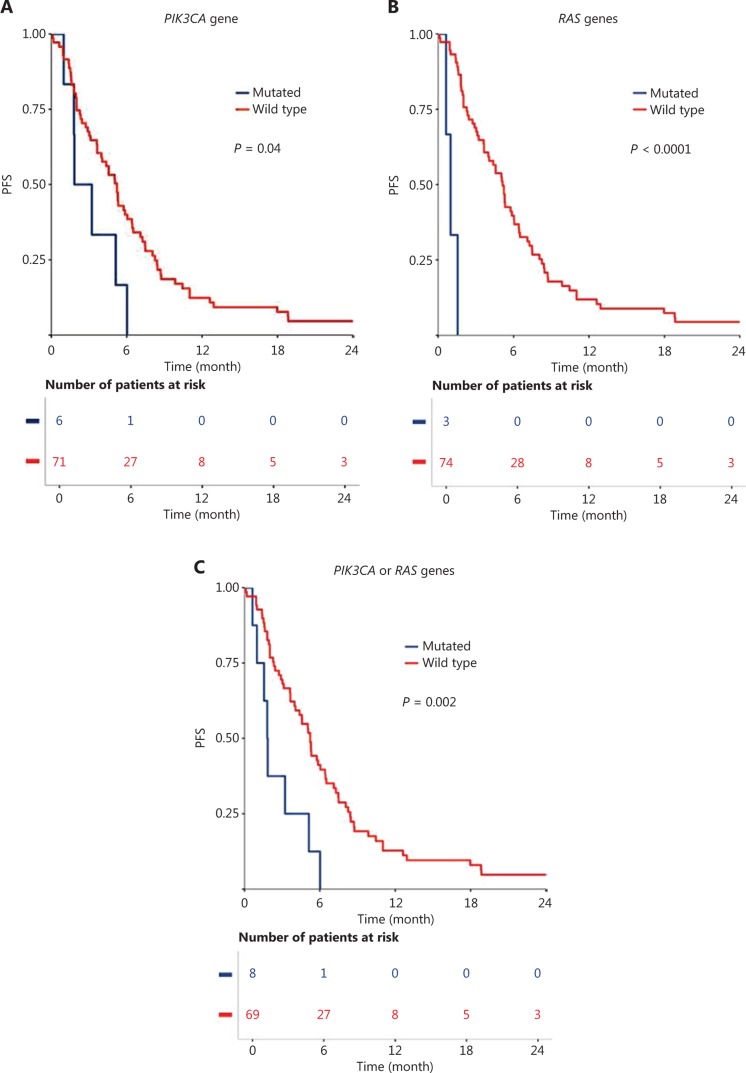
Progression-free survival (PFS) with cetuximab in first-line recurrent and/or metastatic setting, compared between patients with wild-type and oncogene mutations for the following genes: (A) *PIK3CA* gene, (B) *RAS* gene, and (C) *PIK3CA* or *RAS* genes.

Among the 77 HNSCC patients treated with cetuximab in the first-line R/M setting, the median follow-up was 42.7 months (range: 3.2–57.1 months). Within this group, 7.8% had *PIK3CA* mutations, and 3.9% had mutations in *RAS* genes (*KRAS* or *HRAS*) (**Table 2**). *PIK3CA* mutation (*P* = 0.04), *RAS* gene mutation (*P* < 0.001), and all mutations together (*PIK3CA* or *KRAS* or *HRAS*; *P* = 0.002) correlated with a poor PFS (**Table 2**; **Figure 2A, 2B**, and **2C**).

#### Group 2: cetuximab combined with radiotherapy

Among the 38 HNSCC patients treated with cetuximab combined with radiotherapy, the median follow-up was 62.7 months (range: 4.6–92.5 months). In this group, 15.8% of patients showed hot spot activating *PIK3CA* mutations, and 7.9% showed *RAS* gene mutations (**Table 2**). PFS did not significantly differ between patients with and without mutations of the tested oncogenes.

### Predictive value of the loss of PTEN protein expression for cetuximab efficacy

PTEN protein expression loss was detected in 7 patients (6.1%) in the whole population—including 6 patients (7.8%) from group 1, and 1 patient (2.6%) from group 2 (**Figure 1**, **Table 2**). Loss of PTEN protein expression was associated with poor PFS in the whole population, although this association was not statistically significant (*P* = 0.10). The only patient who was treated with cetuximab and radiotherapy and who had a loss of PTEN expression experienced an early recurrence. We observed mutual exclusion between hot spot activating *PIK3CA* or *RAS* mutations and loss of *PTEN* protein expression (**Figure 1**).

### Predictive value of combined oncogene mutations and loss of PTEN protein expression for cetuximab efficacy

Survival analyses suggested a negative predictive value of *PIK3CA* or *RAS* mutational status and for loss of PTEN protein expression for cetuximab efficacy (**Table 2**). At least one resistance biomarker was detected in 24 patients (20.9%) from the whole population—including 14 (18.2%) from group 1, and 10 (26.3%) from group 2 (**Table 2**).

**Figure 3:**
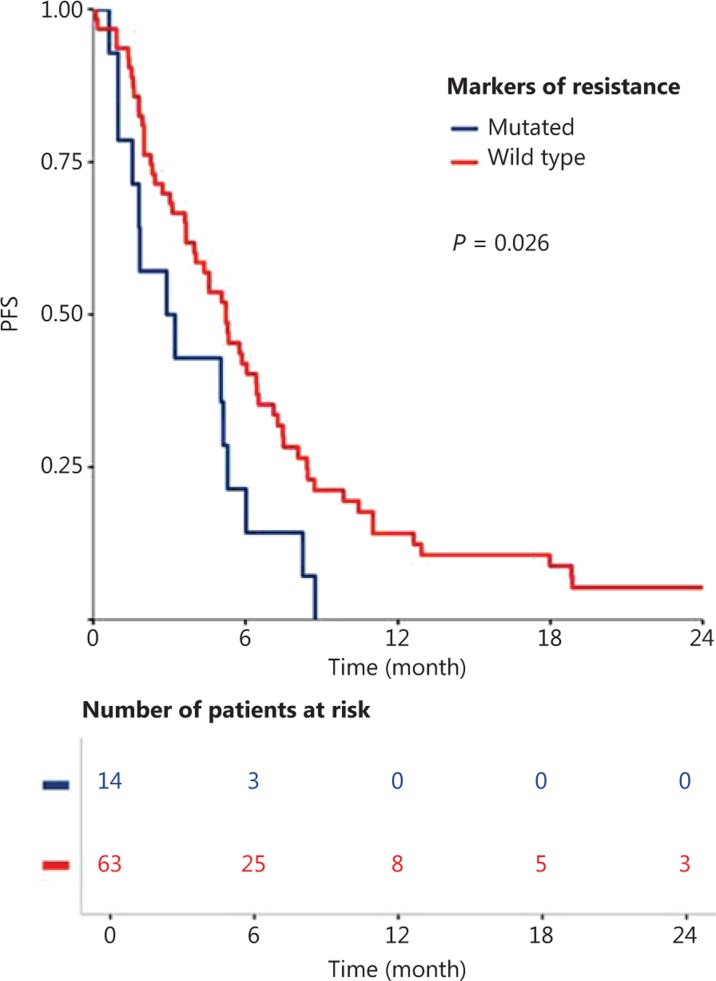
Progression-free-survival (PFS) with cetuximab in first-line recurrence and/or metastatic setting, compared between patients with and without a biomarker of resistance (*PIK3CA* mutation, *RAS* mutation, or loss of PTEN protein expression).

Global resistance biomarker (*PIK3CA/RAS* mutations and PTEN loss) status significantly correlated with PFS among patients treated with cetuximab in the first-line R/M setting (*P* = 0.026; **Figure 3**). These global resistance biomarkers were not significantly correlated with PFS in the subgroup of patients treated with cetuximab alone (**Supplementary Figure S1A**) or cetuximab in combination with chemotherapy (**Supplementary Figure S1B**), however, a tendency towards poor prognosis was observed in the cetuximab-treated patients. In this patient subpopulation (group 1), global resistance biomarker status was not significantly associated with any clinical, biological, or pathological parameters, except for age (**Supplementary Table S5**). Global resistance biomarker status was also found not to be associated with PFS in the whole population, or in the subgroup of patients treated with cetuximab in combination with radiotherapy (group 2; **Supplementary Figure S2**).

Global resistance biomarker status tended to correlate with PFS within the HPV-negative subgroup of patients (**Supplementary Figure S3A**) as compared to HPV-positive patients (**Supplementary Figure S3B**).

### Predictive value of EGFR protein expression for cetuximab efficacy

EGFR protein overexpression was observed in 18 patients (15.7%) in the whole population, including 14 patients in group 1 (18.2%), and 4 patients (10.5%) in group 2 (**Figure 1**, **Table 2**). PFS did not differ significantly according to the EGFR protein level in the whole population, or in the two subgroups (**Table 2**).

## Discussion

HNSCC patients do not undergo any molecular selection prior to treatment with cetuximab, in contrast with metastatic colorectal cancer patients who are required to have a tumor with wild-type *KRAS*^13^. EGFR protein expression was not shown to be predictive of cetuximab efficacy in our patient population. The literature reports that EGFR expression and amplifications are not predictive of EGFR inhibition in HNSCC^17^. The main reason for this is that *RAS* mutations are rare in HNSCC. The PI3K/AKT pathway is frequently activated in HNSCC, and may constitute a source of tumor escape during EGFR targeting^18,19^. Therefore, here we focused on alterations in the PI3K/AKT and RAS/MAPK pathways as potential biomarkers of resistance to cetuximab among the 115 HNSCC patients treated with cetuximab at the Curie Institute.

Our patient population was representative of previously reported HNSCC populations, in which tobacco and HPV are main prognostic factors^2^. Hot spot activating *PIK3CA* and *H/KRAS* mutations were observed in 10.4% and 3.5% of HNSCC patients, respectively, which is also consistent with previous reports^12,13^. IHC revealed loss of PTEN protein expression in 6.1% of our patient population, as compared to a rate of 12% found by RNA sequencing in TCGA^12^.

We demonstrated that hot spot activating *PIK3CA* and *KRAS/HRAS* mutations were associated with poor PFS among HNSCC patients treated with cetuximab in the first-line recurrent setting, but not among patients treated with cetuximab in combination with radiotherapy. We also showed that the loss of PTEN protein expression tended to have a negative prognostic impact in HNSCC patients. EGFR expression did not appear to be a predictive biomarker of cetuximab efficacy, in accordance with previous reports^9^.

In non-small cell lung cancer (NSCLC), EGFR tyrosine kinase inhibitors show limited efficacy over time, with all patients eventually progressing despite treatment with these drugs^20^. Activating *KRAS* mutations and PTEN loss reportedly lead to PI3K/AKT pathway activation, independent of EGFR tyrosine kinase status, driving the downstream cancer survival pathways and supporting resistance^21^. In patients with *HER2*-positive breast cancer, *PIK3CA* mutations reportedly confer resistance to trastuzumab, a monoclonal antibody targeting *HER2*^22^. Additionally, PTEN loss is frequently reported in *HER2*-overexpressing esophageal cancer, and has been associated with poor efficacy of trastuzumab-based therapy^23^. However, studies have reported contradictory data regarding the predictive values of PTEN and PI3K pathway alterations with regards to trastuzumab efficacy^24–26^.

The findings of our group and others suggest that combined therapy is important for overcoming treatment resistance due to PI3K/AKT pathway alterations in HNSCC. Preclinical studies have investigated the combination of EGFR inhibitors and PI3K/AKT pathway inhibitors, showing synergistic effects that induce apoptosis^27^. In phase I clinical trials, the pan-class I PI3K inhibitors pilaralisib (SAR245408, XL147) and SAR245409 (XL765) were safely combined with erlotinib, but exhibited limited antitumor activity^28,29^. A randomized phase II trial demonstrated that combination of the irreversible PI3K inhibitor PX-866 with cetuximab did not improve outcomes among HNSCC patients treated without molecular preselection^30^. Two ongoing trials are evaluating the combination of BKM120 with erlotinib (NCT01487265) and gefitinib (NCT01570296). Another ongoing phase Ib/II trial is testing the efficacy of the PI3K inhibitor copanlisib in combination with cetuximab among patients with recurrent and/or metastatic HNSCCs that harbor a *PIK3CA* mutation/amplification and/or a PTEN loss (NCT02822482). It remains to be demonstrated whether these combinations will successfully overcome primary resistance to EGFR inhibitors alone.

Our present results indicated that hot spot activating *PIK3CA* and *RAS* mutations were biomarkers of cetuximab resistance among HNSCC patients in the first-line recurrence setting, as is also the case in colorectal cancer^14,15^. We also showed that PTEN loss might be a biomarker of resistance to cetuximab when administered in combination with radiotherapy. Of note, as all patients treated with either cetuximab or in combination with chemotherapy were treated in the same clinical setting (i.e. R/M), we decided to pull all these patients together in order to get more statistical power. This might have biased the results as prolonged survival on the combination might either be due to cetuximab alone or to chemotherapy or radiotherapy. These results need to be further validated in independent cohorts taking into account treatment regimens. Overall, our results suggest that alterations in the *PIK3CA, RAS*, and *PTEN* genes may serve as biomarkers for patient selection in future clinical trials involving treatment with cetuximab alone or in combination with other therapies.

## Figures and Tables

**Table 1:** Clinical, biological, and pathological characteristics of the 115 HNSCC patients, and their associations with PFS

Characteristics	*n*	(%)	Progression^c^	HR^d^	95% CI (HR)	PFS^e^
Total	115	(100.0)	97			
Age at diagnosis						0.21
<60 years	53	(46.1)	47	1		
≥60 years	62	(53.9)	50	0.77	[0.52; 1.15]	
Gender						0.14
Female	26	(22.6)	18	1		
Male	89	(77.4)	79	1.47	[0.87; 2.45]	
Tobacco^a^						**0.007**
No	15	(13.6)	8	1		
Yes	95	(86.4)	85	2.63	[1.27; 5.45]	
Alcohol^b^						0.33
No	37	(42.5)	28	1		
Yes	50	(57.5)	45	1.27	[0.79; 2.03]	
HPV status						**0.007**
Negative	100	(87.0)	88	1		
Positive	15	(13.0)	9	0.4	[0.2; 0.8]	
AJCC stage						0.13
Stage I–II	16	(13.9)	14	1		
Stage III	24	(20.9)	17	0.56	[0.27; 1.14]	
Stage IV	75	(65.2)	66	0.94	[0.53; 1.69]	
Tumor location						0.67
Oral cavity	33	(28.7)	29	1		
Oropharynx	50	(45.5)	41	0.74	[0.46; 1.20]	
Larynx	15	(13.0)	12	0.87	[0.44; 1.71]	
Hypopharynx	8	(7.00)	8	1.15	[0.53; 2.53]	
Other	9	(7.80)	7	0.98	[0.43; 2.25]	
Treatment setting				NA^f^	NA^f^	NA^f^
Cetuximab in first-line recurrent setting	77	(67.0)	72			
Cetuximab + radiotherapy	38	(33.0)	25			

Bold values are statistically significant. ^a^Information available for 110 patients; ^b^information available for 87 patients; ^c^progression data collected until June 2016; ^d^HR estimated by non-adjusted Cox proportional hazards model; ^e^*P* value of the log-rank test; ^f^Not applicable as the clinical setting is different. HR, hazard ratio; HNSCC, head and neck squamous cell carcinoma; PFS, progression-free survival.

**Table 2:** Prevalence of DNA mutations and variations in protein expression in the 115 HNSCC samples in relation with PFS

	Whole population (*n* = 115)					Cetuximab in first-line recurrent setting (*n* = 77)					Cetuximab + radiotherapy (*n* = 38)				
	*n* (%)	Progression^a^	HR^d^	95% CI (HR)	PFS^e^	*n* (%)	Progression^a^	HR^d^	95% CI (HR)	PFS^e^	*n* (%)	Progression^a^	HR^d^	95% CI (HR)	PFS^e^
DNA mutations															
*PIK3CA*															
Mutated	12 (10.4)	9	1		0.30	6 (7.8)	6	1		**0.04**	6 (15.8)	3	1		0.22
Wild type	103 (89.6)	88	1.45	[0.72; 2.89]		71 (92.2)	66	0.42	[0.18; 0.99]		32 (84.2)	22	2.12	[0.63; 7.14]	
All RAS															
Mutated	6 (5.2)	5	1		0.96	3 (3.9)	3	1		**<0.001**	3 (7.9)	2	1		0.69
Wild type	109 (94.8)	92	1.02	[0.41; 2.52]		74 (96.1)	69	0.07	[0.02; 0.26]		35 (92.1)	23	1.34	[0.31; 5.68]	
All mutations															
Mutated	17^b^(14.8)	13	1		0.23	8^b^(10.4)	8	1		**0.002**	9 (23.7)	5	1		0.17
Wild type	98 (85.2)	84	1.43	[0.79; 2.59]		69 (89.6)	64	0.32	[0.15; 0.7]		29 (76.3)	20	1.96	[0.73; 5.29]	
Level of protein expression															
PTEN^c^															
Low	7 (6.1)	7	1		0.10	6 (7.8)	6	1		0.71	1 (2.6)	1	1		**0.02**
High	107 (93.0)	89	0.52	[0.24; 1.14]		71 (92.2)	66	0.85	[0.37; 1.98]		36 (94.7)	23	0.12	[0.01; 1.05]	
*EGFR*															
Low	97 (84.3)	81	1		0.62	63 (81.8)	60	1		0.24	34 (89.5)	21	1		0.08
High	18 (15.7)	16	1.15	[0.67; 1.97]		14 (18.2)	12	0.69	[0.37; 1.29]		4 (10.5)	4	2.55	[0.85; 7.64]	

Bold values are statistically significant. ^a^Progression data collected until June 2016; ^b^one patient has both *PIK3CA* and *HRAS* mutations; ^c^one patient has not been tested; ^d^HR estimated by non-adjusted Cox proportional hazards model; ^e^*P*-value of the log-rank test. HR, hazard ratio; HNSCC, head and neck squamous cell carcinoma; PFS, progression-free survival.

## References

[1] Ferlay J, Soerjomataram I, Dikshit R, Eser S, Mathers C, Rebelo M (2015). Cancer incidence and mortality worldwide: sources, methods and major patterns in GLOBOCAN 2012.. Int J Cancer..

[2] Ang KK, Harris J, Wheeler R, Weber R, Rosenthal DI, Nguyen-Tân PF (1993). Human papillomavirus and survival of patients with oropharyngeal cancer.. N Engl J Med..

[3] Bonner JA, Harari PM, Giralt J, Azarnia N, Shin DM, Cohen RB (1993). Radiotherapy plus cetuximab for squamous-cell carcinoma of the head and neck.. N Engl J Med..

[4] Vermorken JB, Mesia R, Rivera F, Remenar E, Kawecki A, Rottey S (1993). Platinum-based chemotherapy plus cetuximab in head and neck cancer.. N Engl J Med..

[5] Depenni R, Cossu Rocca M, Ferrari D, Azzarello G, Baldessari C, Alù M (1993). Clinical outcomes and prognostic factors in recurrent and/or metastatic head and neck cancer patients treated with chemotherapy plus cetuximab as first-line therapy in a real-world setting.. Eur J Cancer..

[6] Byrne K, Hallworth P, Monfared AAT, Moshyk A, Shaw JW (2019). Real-world systemic therapy treatment patterns for squamous cell carcinoma of the head and neck in Canada.. Curr Oncol..

[7] Nadler E, Joo S, Boyd M, Black-Shinn J, Chirovsky D (1993). Treatment patterns and outcomes among patients with recurrent/metastatic squamous cell carcinoma of the head and neck.. Future Oncol..

[8] Byrne K, Zanotti G, Hallworth P, Roughley A, Martini JF, Uehara R (1993). Real-world treatment patterns and outcomes of patients with stage IV squamous cell carcinoma of the head and neck.. Future Oncol..

[9] Licitra L, Mesia R, Rivera F, Remenár É,  Hitt R, Erfán J (1993). Evaluation of *EGFR* gene copy number as a predictive biomarker for the efficacy of cetuximab in combination with chemotherapy in the first-line treatment of recurrent and/or metastatic squamous cell carcinoma of the head and neck: EXTREME study.. Ann Oncol..

[10] Bertotti A, Sassi F (1993). Molecular pathways: sensitivity and resistance to anti-EGFR antibodies.. Clin Cancer Res..

[11] Bossi P, Bergamini C, Siano M, Cossu Rocca M, Sponghini AP, Favales F (1993). Functional genomics uncover the biology behind the responsiveness of head and neck squamous cell cancer patients to cetuximab.. Clin Cancer Res..

[12] The Cancer Genome Atlas Network (1993). Comprehensive genomic characterization of head and neck squamous cell carcinomas.. Nature..

[13] Sablin MP, Dubot C, Klijanienko J, Vacher S, Ouafi L, Chemlali W (1993). Identification of new candidate therapeutic target genes in head and neck squamous cell carcinomas.. Oncotarget..

[14] Karapetis CS, Khambata-Ford S, Jonker DJ, O’Callaghan CJ, Tu DS, Tebbutt NC (1993). *K-ras* mutations and benefit from cetuximab in advanced colorectal cancer.. N Engl J Med..

[15] Lièvre A, Bachet JB, Le Corre D, Boige V, Landi B, Emile JF (1993). KRAS mutation status is predictive of response to cetuximab therapy in colorectal cancer.. Cancer Res..

[16] de la Rochefordiere A, Kamal M, Floquet A, Thomas L, Petrow P, Petit T (1993). *PIK3CA* pathway mutations predictive of poor response following standard radiochemotherapy ± cetuximab in cervical cancer patients.. Clin Cancer Res..

[17] Bossi P, Resteghini C, Paielli N, Licitra L, Pilotti S, Perrone F (1993). Prognostic and predictive value of EGFR in head and neck squamous cell carcinoma.. Oncotarget..

[18] García-Carracedo D, Villaronga MÁ, Álvarez-Teijeiro S, Hermida-Prado F, Santamaría I, Allonca E (1993). Impact of PI3K/AKT/mTOR pathway activation on the prognosis of patients with head and neck squamous cell carcinomas.. Oncotarget..

[19] Iglesias-Bartolome R, Martin D, Gutkind JS (1993). Exploiting the head and neck cancer oncogenome: widespread PI3K-mTOR pathway alterations and novel molecular targets.. Cancer Discov..

[20] Rosell R, Moran T, Queralt C, Porta R, Cardenal F, Camps C (1993). Screening for epidermal growth factor receptor mutations in lung cancer.. N Engl J Med..

[21] Morgillo F, Della Corte CM, Fasano M, Ciardiello F (2016). Mechanisms of resistance to EGFR-targeted drugs: lung cancer.. ESMO Open..

[22] Cizkova M, Dujaric ME, Lehmann-Che J, Scott V, Tembo O, Asselain B (1993). Outcome impact of *PIK3CA* mutations in HER2-positive breast cancer patients treated with trastuzumab.. Br J Cancer..

[23] Deguchi Y, Okabe H, Oshima N, Hisamori S, Minamiguchi S, Muto M (1993). PTEN loss is associated with a poor response to trastuzumab in HER2-overexpressing gastroesophageal adenocarcinoma.. Gastric Cancer..

[24] Nagata Y, Lan KH, Zhou XY, Tan M, Esteva FJ, Sahin AA (1993). PTEN activation contributes to tumor inhibition by trastuzumab, and loss of PTEN predicts trastuzumab resistance in patients.. Cancer Cell..

[25] Berns K, Horlings HM, Hennessy BT, Madiredjo M, Hijmans EM, Beelen K (2007). A functional genetic approach identifies the PI3K pathway as a major determinant of trastuzumab resistance in breast cancer.. Cancer Cell..

[26] Esteva FJ, Guo H, Zhang SY, Santa-Maria C, Stone S, Lanchbury JS (1993). PTEN, PIK3CA, p-AKT, and p-p70S6K status: association with trastuzumab response and survival in patients with HER2-positive metastatic breast cancer.. Am J Pathol..

[27] Dave B, Migliaccio I, Gutierrez MC, Wu MF, Chamness GC, Wong H (1993). Loss of phosphatase and tensin homolog or phosphoinositol-3 kinase activation and response to trastuzumab or lapatinib in human epidermal growth factor receptor 2-overexpressing locally advanced breast cancers.. J Clin Oncol..

[28] Soria JC, LoRusso P, Bahleda R, Lager J, Liu L, Jiang J (1993). Phase I dose-escalation study of pilaralisib (SAR245408, XL147), a pan-class I PI3K inhibitor, in combination with erlotinib in patients with solid tumors.. Oncologist..

[29] Jänne PA, Cohen RB, Laird AD, Macé S, Engelman JA, Ruiz-Soto R (1993). Phase I safety and pharmacokinetic study of the PI3K/mTOR inhibitor SAR245409 (XL765) in combination with erlotinib in patients with advanced solid tumors.. J Thorac Oncol..

[30] Jimeno A, Shirai K, Choi M, Laskin J, Kochenderfer M, Spira A (1993). A randomized, phase II trial of cetuximab with or without PX-866, an irreversible oral phosphatidylinositol 3-kinase inhibitor, in patients with relapsed or metastatic head and neck squamous cell cancer.. Ann Oncol..

