# Supplementary material for: Biomarkers of cetuximab resistance in patients with head and neck squamous cell carcinoma
Source: Cancer Biol Med. 2020 Feb 15;17(1):208–17. doi: 10.20892/j.issn.2095-3941.2019.0153 (PMC7142836; doi:10.20892/j.issn.2095-3941.2019.0153)
Supplement: Supplementary file 1 [file cbm-17-208-s001.pdf]

Supplementary materials

Table S1 Clinical, biological, and pathological characteristics of the 77 HNSCC patients who were treated with cetuximab in the first-line recurrent and/or metastatic setting, and their associations with PFS

Characteristics	*n* (%)	Progression[c]	HR[d]	95% CI (HR)	PFS[e]
Total	77 (100.0)	72			
Age at diagnosis					
< 60 years	42 (54.5)	40	1		0.51
≥ 60 years	35 (45.5)	32	1.17	[0.73; 1.87]	
Gender					
Female	18 (23.4)	14	1		0.74
Male	59 (76.6)	58	1.11	[0.61; 2]	
Tobacco[a]					
No	8 (89.0)	6	1		0.38
Yes	65 (11.0)	63	1.45	[0.63; 3.37]	
Alcohol[b]					
No	24 (43.6)	21	1		0.93
Yes	31 (56.4)	31	0.97	[0.55; 1.72]	
HPV status					
Negative	71 (92.2)	66	1		0.32
Positive	6 (7.8)	6	1.53	[0.66; 3.57]	
AJCC stage					
Stage I–II	13 (16.9)	11	1		0.16
Stage III	12 (15.6)	11	1.57	[0.68; 3.63]	
Stage IV	52 (67.5)	50	1.88	[0.97; 3.62]	
Tumor location					
Oral cavity	28 (36.4)	24	1		0.47
Oropharynx	27 (35.0)	27	1.30	[0.74; 2.26]	
Larynx	10 (13.0)	10	1.95	[0.91; 4.14]	
Hyopharynx	7 (9.1)	7	1.30	[0.56; 3.04]	
Other	5 (6.5)	4	1.76	[0.61; 5.14]	

[a]Information available for 73 patients; [b]information available for 55 patients; [c]progression data collected until June 2016; [d]HR estimated by non-adjusted Cox proportional hazards model; [e]*P* value of the log-rank test. AJCC; American Joint Committee on Cancer; CI, confidence interval; HR, hazard ratio; HNSCC, head and neck squamous cell carcinoma; HPV; human papillomavirus; PFS, progression-free survival.

Table S2 Clinical, biological, and pathological characteristics of the 38 HNSCC patients who were treated with cetuximab combined with radiotherapy, and their associations with PFS

Characteristics	*n* (%)	Progression[c]	HR[d]	95% CI (HR)	PFS[e]
Total	38 (100.0)	25			
Age at diagnosis					
< 60 years	11 (28.9)	7	1		0.89
≥ 60 years	27 (71.1)	18	0.94	[0.39; 2.25]	
Gender					
Female	8 (21.1)	4	1		0.13
Male	30 (78.9)	21	2.25	[0.77; 6.58]	
Tobacco[a]					
No	7 (18.9)	2	1		**0.03**
Yes	30 (81.1)	22	4.34	[1.02; 18.55]	
Alcohol[b]					
No	13 (40.6)	7	1		0.19
Yes	19 (59.4)	14	1.82	[0.73; 4.52]	
HPV status					
Negative	29 (76.3)	22	1		**0.01**
Positive	9 (23.7)	3	0.23	[0.07; 0.78]	
AJCC stage					
Stage I–II	3 (7.9)	3	1		**<0.001**
Stage III	12 (31.6)	6	0.02	[0; 0.16]	
Stage IV	23 (60.5)	16	0.04	[0.01; 0.23]	
Tumor location					
Oral cavity	5 (13.2)	5	1		0.58
Oropharynx	23 (60.5)	14	0.53	[0.19; 1.47]	
Larynx	5 (13.2)	2	0.31	[0.06; 1.61]	
Hypopharynx	1 (2.6)	1	0.61	[0.07; 5.25]	
Other	4 (10.5)	3	0.85	[0.20; 3.57]	

[a]Information available for 37 patients; [b]information available for 32 patients; [c]progression data collected until June 2016; [d]HR estimated by non-adjusted Cox proportional hazards model; [e]*P* value of the log-rank test. AJCC; American Joint Committee on Cancer; CI, confidence interval; HR, hazard ratio; HNSCC, head and neck squamous cell carcinoma; HPV; human papillomavirus; PFS, progression-free survival.

Table S3 Clinical, biological, and pathological characteristics of the HNSCC patients, compared between those treated with cetuximab in the locally advanced setting versus in an R/M setting

Characteristics	Cetuximab in first line recurrent and/or metastatic setting (group 1) (%)	RT + cetuximab (group 2) (%)	*P*[c]
Total	77 (100.0)	38 (100.0)	
Age			
< 60 years	42 (54.5)	11 (28.9)	**0.01**
≥ 60 years	35 (45.5)	27 (71.1)	
Gender			
Male	59 (76.6)	30 (78.9)	0.78
Female	18 (23.4)	8 (21.1)	
Tobacco[a]			
Yes	65 (89.0)	30 (81.1)	0.25
No	8 (11.0)	7 (18.9)	
Alcohol[b]			
Yes	31 (56.4)	19 (59.4)	0.78
No	24 (43.6)	13 (40.6)	
HPV status			
Positive	6 (7.8)	9 (23.7)	**0.04**
Negative	71 (92.2)	29 (76.3)	
AJCC stage			
Stage I–II	13 (16.9)	3 (7.9)	0.09
Stage III	12 (15.6)	12 (31.6)	
Stage IV	52 (67.5)	23 (60.5)	
Tumor location			
Oral cavity	28 (36.4)	5 (13.2)	**0.03**
Oropharynx	27 (35.0)	23 (60.5)	
Larynx	10 (13)	5 (13.2)	
Hypopharynx	7 (9.1)	1 (2.6)	
Other	5 (6.5)	4 (10.5)	

[a]Information available for 110 patients (73 patients for group 1, 37 for group 2); [b]information available for 87 patients (55 patients for group 1, 32 for group 2); [c]Yates's χ^2 test. AJCC; American Joint Committee on Cancer; CT, chemotherapy; CI, confidence interval; HR, hazard ratio; HNSCC, head and neck squamous cell carcinoma; HPV; human papillomavirus; PFS, progression-free survival; R/M, recurrent and/or metastatic; RT, radiotherapy.

Table S4 Mutational profiles of the 115 HNSCC samples

Gene	Exon	Mutation status	Number of mutated samples
PIK3CA	9	p.E545K; c.1633G>A	5
		p.E542K; c.1624G>A	3
	20	p.H1047R; c.3140A>G	4
KRAS	2	p.G12C; c.34G>T	1
	4	p.A146V; c.437C>T	1
HRAS	2	p.G12D; c.35G>A	1
		p.G12S; c.34G>A	1
	3	p.Q61R; c.182A>G	1
		p.Q61L; c.182A>T	1

HNSCC, head abd neck squamous cell carcinoma.

Table S5 Relationship between global resistance biomarker status and the clinical, biological, and pathological parameters among the 77 HNSCC patients treated with cetuximab in first-line recurrent and/or metastatic setting

Characteristics	Tumors with a resistance biomarker (%)	Tumors without a resistance biomarker (%)	P^c
Total	14 (18.2)	63 (81.8)	
Age			
< 60 years	4 (28.6)	38 (60.3)	**0.03**
≥ 60 years	10 (71.4)	25 (39.7)	
Gender			
Male	11 (78.6)	48 (76.2)	0.85
Female	3 (21.4)	15 (23.8)	
Tobacco[a]			
Yes	11 (91.7)	54 (88.5)	0.75
No	1 (8.3)	7 (11.5)	
Alcohol[b]			
Yes	6 (54.5)	25 (56.8)	0.89
No	5 (45.5)	19 (43.2)	
HPV status			
Positive	3 (21.4)	3 (4.8)	0.12[c]
Negative	11 (78.6)	60 (95.2)	
AJCC stage			
Stage I–II	1 (7.1)	12 (19.0)	0.28[c]
Stage III	1 (7.1)	11 (17.5)	
Stage IV	12 (85.7)	40 (63.5)	
Tumor location			
Oral cavity	4 (28.6)	24 (38.1)	0.15
Oropharynx	5 (35.7)	22 (34.9)	
Larynx	1 (7.1)	9 (14.3)	
Hypopharynx	1 (7.1)	6 (9.5)	
Other	3 (21.4)	2 (3.2)	

[a]Information available for 73 patients; [b]information available for 55 patients; [c]Yates's χ^2 test. AJCC; American Joint Committee on Cancer; HNSCC, head and neck squamous cell carcinoma; HPV; human papillomavirus.

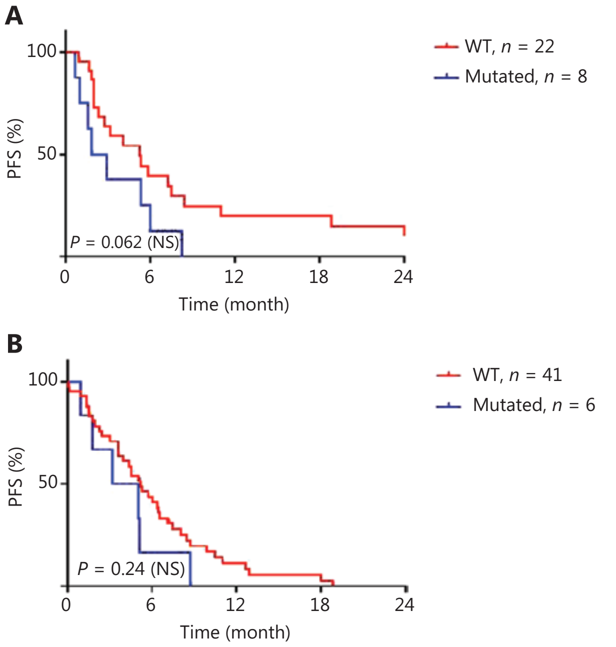

Figure S1 Progression-free-survival (PFS) in a first-line recurrence and/or metastatic setting, comparing patients with and without a biomarker of resistance (*PIK3CA* mutation, *RAS* mutation, or loss of PTEN protein expression). (A) Patients treated with cetuximab alone. (B) Patients treated with cetuximab + chemotherapy.

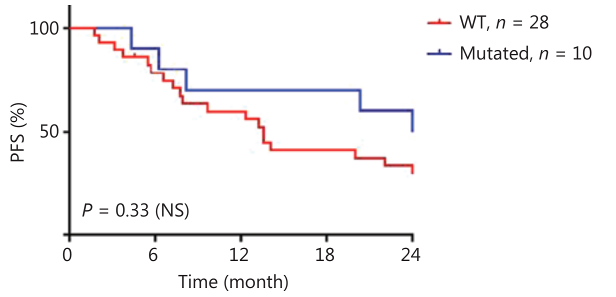

Figure S2 Progression-free-survival (PFS) in group 2 (cetuximab + radiotherapy), comparing patients with and without a biomarker of resistance (*PIK3CA* mutation, *RAS* mutation, or loss of PTEN protein expression).

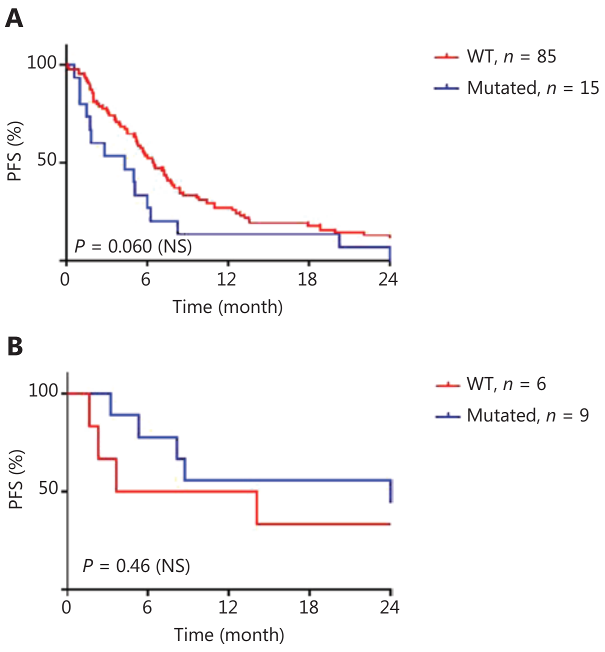

Figure S3 Progression-free-survival (PFS) in patients with and without a biomarker of resistance (PIK3CA mutation, RAS mutation, or loss of PTEN protein expression). (A) human papillomavirus (HPV) negative patients. (B) HPV positive patients.
